# Reactive Oxygen and Nitrogen Species in Defense/Stress Responses Activated by Chitosan in Sycamore Cultured Cells

**DOI:** 10.3390/ijms16023019

**Published:** 2015-01-29

**Authors:** Massimo Malerba, Raffaella Cerana

**Affiliations:** 1 Dipartimento di Biotecnologie e Bioscienze, Università degli Studi di Milano-Bicocca, Piazza della Scienza 2, Milan 20126, Italy; 2 Dipartimento di Scienze dell’Ambiente e del Territorio e di Scienze della Terra, Università degli Studi di Milano-Bicocca, Piazza della Scienza 1, Milan 20126, Italy; E-Mail: raffaella.cerana@unimib.it

**Keywords:** *Acer pseudoplatanus* L., cell death, chitosan, defense response, reactive oxygen species (ROS), reactive nitrogen species (RNS), stress

## Abstract

Chitosan (CHT) is a non-toxic and inexpensive compound obtained by deacetylation of chitin, the main component of the exoskeleton of arthropods as well as of the cell walls of many fungi. In agriculture CHT is used to control numerous diseases on various horticultural commodities but, although different mechanisms have been proposed, the exact mode of action of CHT is still unknown. In sycamore (*Acer pseudoplatanus* L.) cultured cells, CHT induces a set of defense/stress responses that includes production of H_2_O_2_ and nitric oxide (NO). We investigated the possible signaling role of these reactive molecules in some CHT-induced responses by means of inhibitors of production and/or scavengers. The results show that both reactive nitrogen and oxygen species are not only a mere symptom of stress conditions but are involved in the responses induced by CHT in sycamore cells. In particular, NO appears to be involved in a cell death form induced by CHT that shows apoptotic features like DNA fragmentation, increase in caspase-3-like activity and release of cytochrome *c* from the mitochondrion. On the contrary, reactive oxygen species (ROS) appear involved in a cell death form induced by CHT that does not show these apoptotic features but presents increase in lipid peroxidation.

## 1. Introduction

Chitosan (CHT) is a natural, non-toxic and inexpensive compound obtained by partial alkaline deacetylation of chitin, the main component of the exoskeleton of crustaceans and other arthropods as well as of the cell walls of many fungi [1]. Chemically, CHT is a linear, unbranched polymer of β-1,4-d-glucosamine. The variable number of amino groups is very important for its biological activity and makes this polymer very useful for a wide range of industries such as cosmetology (lotions, hair additives, facial and body creams), food (coating, preservative, antioxidant, antimicrobial), biotechnology (chelator, emulsifier, flocculent), pharmacology and medicine (fibers, fabrics, drugs, membranes, artificial organs) and agriculture (soil modifier, films, fungicide, elicitor) [2].

In agriculture CHT has been shown to be a versatile non-toxic compound that controls numerous pre- and post-harvest diseases on various horticultural commodities [2]. To date, there is enough evidence indicating that CHT application makes plants more tolerant to a wide variety of both soil and foliar pathogens like fungi, bacteria, and viruses [3]. This effect, together with the observed induction of root nodulation by CHT [4], proposes this natural compound as a useful tool in the goal of sustainable agriculture. The CHT protective effect can be observed at different levels. In fact, CHT has a direct effect on the morphology of the microorganism, induces the synthesis of structural barriers (suberization and lignification) at the site of attempted pathogen penetration, and can act as an exogenous elicitor of host defense responses when applied to plant tissues or cultured plant cells. The reported defense responses elicited by CHT include: raising of cytosolic Ca^2+^ [5], activation of MAP kinases [6], callose apposition [7], oxidative burst [8], cell death near the site of infection to limit the diffusion of the pathogen (the so called hypersensitive response, HR) [9], synthesis of abscisic acid (ABA), jasmonate, pathogenesis related proteins (PR), and phytoalexins [10,11].

Although different mechanisms have been proposed, the exact mode of action of CHT is still unknown. It has been proposed that the interaction between positively charged CHT molecules and the negatively charged hydrophilic portion of phospholipids of microbial cell plasma membrane may lead to the leakage of proteinaceous and other intracellular constituents. CHT may also act as a chelating agent that selectively binds trace metals and thereby inhibits the production of toxins and microbial growth. Interestingly, CHT can reach the nuclei of pathogens, breaking DNA strands and removing histones H2A and H2B [12,13]. These direct CHT/DNA interactions can influence the transcription of pathogenesis-related (PR) gene mRNA and PR protein synthesis [14].

On the basis of the mechanism of action of other elicitors, the possible presence of specific receptors for CHT has been investigated [10], and the results strongly suggest the presence of putative CHT receptors and encourage further studies to clarify the signal transduction pathway leading to the responses induced by CHT treatment [15].

With some restrictions, plant cell cultures represent a useful system to study the responses to exogenous compounds as they are formed by more homogeneous cells than those present in complex tissues. In addition the administration of compounds and the reproducibility of the experimental conditions are easy in this more controlled system.

In sycamore (*Acer pseudoplatanus* L.) cultured cells, a material well characterized both biochemically and physiologically, CHT rapidly induces a set of defense/stress responses: cell death that in a fraction of dead cells show apoptotic features like DNA fragmentation and release of cytochrome *c* from the mitochondrion, production of H_2_O_2_ and nitric oxide (NO), accumulation of regulative 14-3-3 proteins in the cytosol and of heat-shock protein 70 (HSP70) molecular chaperone Binding Protein (BiP) in the endoplasmic reticulum [16]. These findings, in particular the production of H_2_O_2_ and NO induced by CHT, open the possibility to use sycamore cultured cells to study the signaling pathways leading to the CHT-induced responses. In addition, sycamore is an economically important plant and although good genomic information is still lacking in this plant species, it is known for its ability to tolerate wind, salt spray and urban pollution. This makes sycamore a popular tree for planting in coastal towns and along city roads treated with salt in winter.

Reactive oxygen and nitrogen species (ROS and RNS), that result from incomplete reduction and excitation processes, are harmful by-products of cellular metabolism in aerobic organisms. Due to their peculiar metabolism, plants in particular must face an even greater excess of ROS and RNS [17]. Thus, these organisms developed various protective enzymatic and non-enzymatic mechanisms to keep reactive species under control. As these protective mechanisms became very efficient, plants further evolved a very elaborate network of enzymes to purposefully adjust ROS and RNS levels in different cell types and organs at different times and developmental stages. This evolution permitted the adaptive cooptation of ROS and RNS as signaling molecules in different plant processes. In addition to their involvement in plant development, ROS and RNS, in particular H_2_O_2_ and NO, can act as second messengers during plant responses to several abiotic and biotic stresses that include salinity, drought, ultraviolet radiation, temperature, heavy metals, and pathogen attack [18]. In particular, the role of these reactive molecular species in regulating hypersensitive response associated with pathogen interaction is very well established. Further, H_2_O_2_ and NO are known to activate the transcriptional factors of pathogenesis-related proteins during the induction of resistance, playing an important role in avoiding pathogen advancement [19,20]. In this work we investigated the possible signaling role of reactive oxygen and nitrogen species in the CHT-induced responses by means of inhibitors of production and/or scavengers. In particular, sycamore cultured cells were challenged with CHT in the absence or in the presence of cPTIO (2-(4-carboxyphenyl)-4,4,5,5-tetramethylimidazoline-1-oxyl-3-oxide), a specific NO scavenger, Tiron (4,5-dihydroxy-1,3-benzene disulfonic acid), a specific scavenger of superoxide anion (O_2_•^−^), and DPI (diphenylene iodonium), a specific inhibitor of the NADPH oxidase (O_2_•^−^ synthase) of the plasma membrane. In these experimental conditions we measured: production of NO, O_2_•^−^ and H_2_O_2_; cell death; accumulation of cells with fragmented DNA; release of cytochrome *c* from the mitochondrion; level of lipid peroxidation; activity of caspase-3-like proteases; accumulation of regulative 14-3-3 proteins in the cytosol and of HSP70 molecular chaperone BiP in the endoplasmic reticulum (ER).

## 2. Results and Discussion

### 2.1. Effect of cPTIO on the CHT-Induced NO Accumulation in the Cells and of Tiron and DPI on the CHT-Induced O_2_•^−^ and H_2_O_2_ Accumulations in the Culture Medium

To explore the possible use of inhibitors of production and/or scavengers to elucidate the role played by reactive oxygen and nitrogen species in the CHT-induced responses, we preliminarily tested the effect of cPTIO on the CHT-induced NO accumulation in the cells and of Tiron and DPI on the CHT-induced O_2_•^−^ and H_2_O_2_ accumulations in the culture medium.

**Figure 1:**
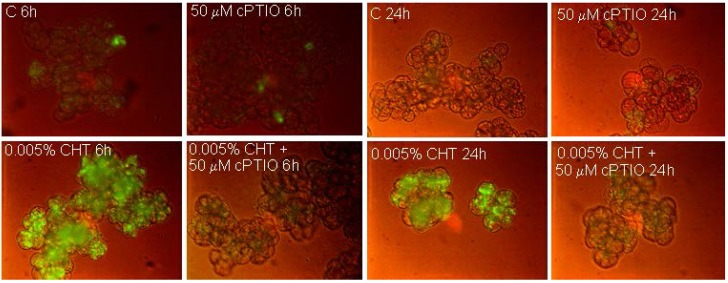
Imaging of NO by fluorescence microscopy using DAF-FM diacetate. At the indicated times, 6 and 24 h after CHT treatment, the cells were collected and stained for NO visualization. C: Control cells; cPTIO: 2-(4-Carboxyphenyl)-4,4,5,5-tetramethylimidazoline-1-oxyl-3-oxide); CHT: Chitosan.

**Figure 2:**
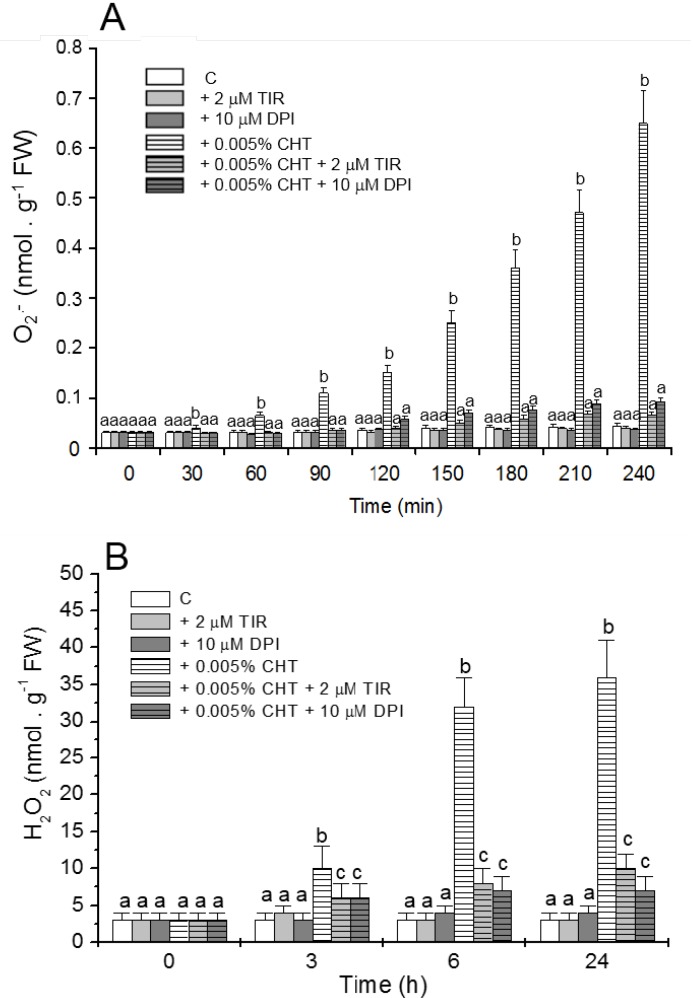
Effect of Tiron (TIR) and diphenylene iodonium (DPI) on the CHT-induced O_2_•^−^ (**A**) and H_2_O_2_ (**B**) accumulations in the culture medium*.* At the indicated times aliquots of culture medium were collected and O_2_•^−^ and H_2_O_2_ content was measured. Mean values ± SD (*n* ≥ 9) are presented. Different letters indicate significant differences among treatments at each experimental time (Tukey HSD test, *p* ≤ 0.05).

NO production in the cells was evaluated by using the specific fluorochrome DAF-FM diacetate that after reaction with NO emits a bright green fluorescence. Figure 1 shows that, as already reported [11] CHT induces the appearance of cells showing bright fluorescence due to NO accumulation. Interestingly, at both experimental times (6 and 24 h), this fluorescence is totally absent in the cells treated with CHT in the presence of cPTIO. Figure 2 shows (see also [11] for H_2_O_2_ accumulation) that CHT induces accumulation of ROS, namely O_2_•^−^ (Figure 2A) and H_2_O_2_ (Figure 2B), and that this accumulation is largely reduced by Tiron and DPI. These results suggest the compounds might be useful to elucidate the role of NO and ROS in the CHT-induced responses of sycamore cells.

### 2.2. Effect of cPTIO, Tiron and DPI on CHT-Induced Cell Death and DNA Fragmentation

**Figure 3:**
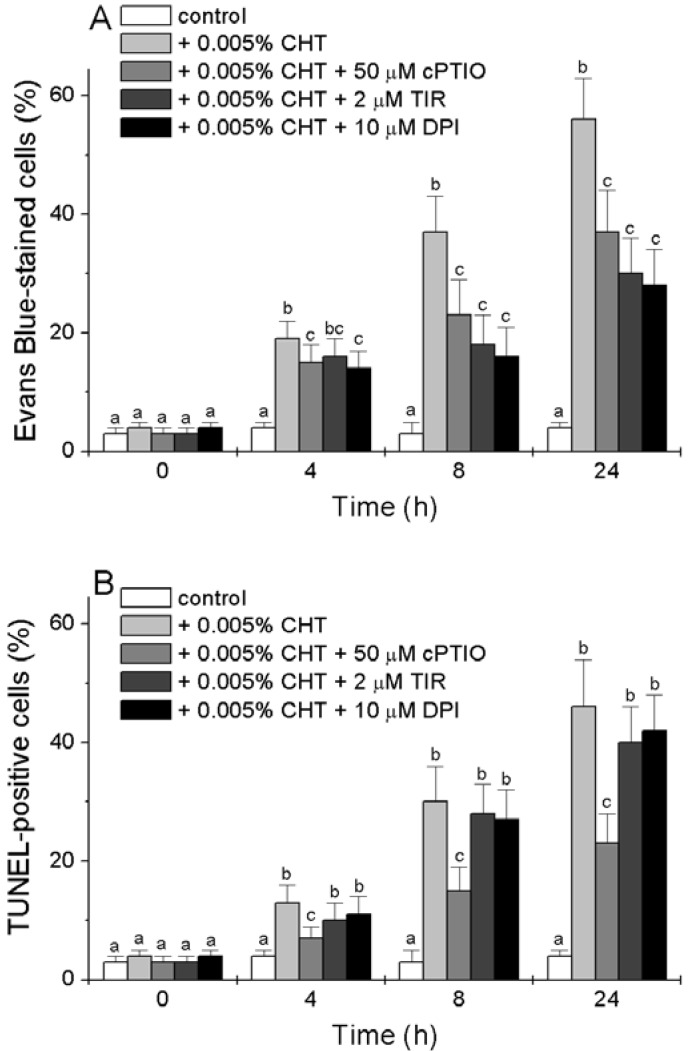
Effect of cPTIO, Tiron and DPI on the CHT-induced cell death (**A**) and DNA fragmentation (**B**). At the indicated times, the cells were collected and stained with Evan’s Blue (**A**) or fixed and subjected to the TUNEL procedure (**B**). Mean values ± SD (*n* ≥ 6) are presented. Different letters indicate significant differences among treatments at each experimental time (Tukey HSD test, *p* ≤ 0.05).

We tested the effect of cPTIO, Tiron and DPI on the CHT-induced accumulation of dead cells and of cells with fragmented DNA. Cell death was determined by Evan’s Blue staining and nuclear DNA fragmentation, a marker of cells undergoing programmed cell death (PCD) with apoptotic features [21] was detected *in situ* by the TUNEL reaction. Figure 3A reconfirms that CHT causes the accumulation of dead cells [16] and shows that this accumulation is reduced by the three chemicals. Tiron and DPI are more efficient than cPTIO, with a 50% reduction in the CHT-induced accumulation of dead cells 8 and 24 h after treatment.

The results presented in Figure 3B show [16] that CHT also induces the appearance of TUNEL-positive cells. At all experimental times the percentage of TUNEL-positive cells is slightly lower than that of dead cells. Interestingly, while the CHT-induced accumulation of TUNEL-positive cells is markedly reduced by cPTIO, both Tiron and DPI are unable to substantially reduce this accumulation.

### 2.3. Effect of CHT, cPTIO, Tiron and DPI on Caspase-3-Like Activity

**Figure 4:**
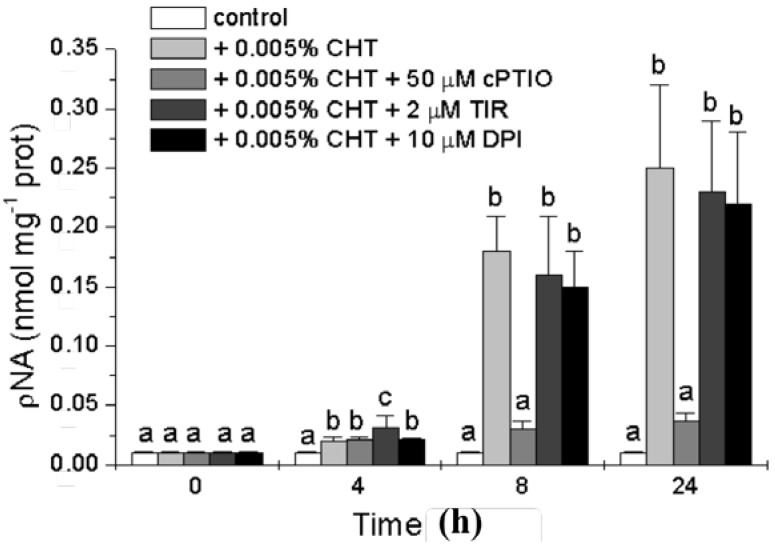
Effect of CHT, cPTIO, Tiron, and DPI on caspase-3-like activity. Mean values ± SD (*n* ≥ 6) are presented. Different letters indicate significant differences among treatments at each experimental time (Tukey HSD test, *p* ≤ 0.05).

To further characterize the nature of the cell death process induced by CHT and to investigate the possible role of the signaling molecules in this process we analyzed the occurrence of another PCD hallmark, the activity of caspase-3-like proteases. The activation of caspase-3-like proteases has been shown to occur during PCD in several plant systems suggesting that some forms of plant PCD may have a caspase triggering pathway similar to the animal counterpart [22]. The activity of caspase-3-like proteases was measured with a colorimetric assay kit (see Experimental Section), and the results presented in Figure 4 show that CHT induces a strong increase in this activity that is markedly reduced by cPTIO. On the contrary, both Tiron and DPI are practically ineffective.

### 2.4. Effect of CHT, cPTIO, Tiron and DPI on the Level of Lipid Peroxidation

**Figure 5:**
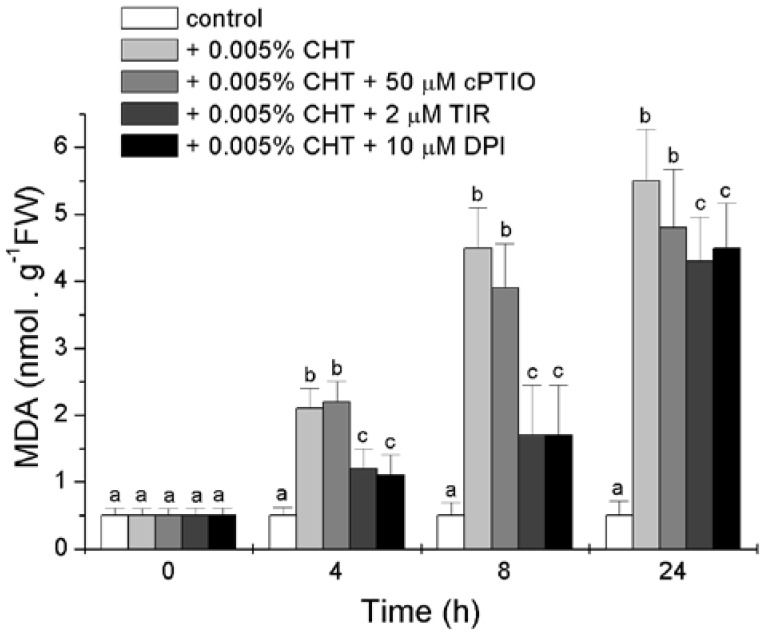
Effect of CHT, cPTIO, Tiron and DPI on the level of lipid peroxidation. Mean values ± SD (*n* ≥ 9) are presented. Different letters indicate significant differences among treatments at each experimental time (Tukey HSD test, *p* ≤ 0.05). MDA: malondialdehyde.

Lipid peroxidation, in both cellular and organellar membranes, is one of the most dangerous consequence of oxidative stress [23]. The level of lipid peroxidation was estimated by measuring the content of malondialdehyde (MDA), a secondary end product of the oxidation of polyunsaturated fatty acids, and the results presented in Figure 5 show that CHT induces a strong increase in the level of MDA. This increase is markedly prevented by Tiron and DPI, at least at the first two experimental times, while cPTIO is less effective. 

### 2.5. Effect of cPTIO, Tiron and DPI on the CHT-Induced Release of Cytochrome c from Mitochondria and Increase in the Levels of Cytosolic 14-3-3 Proteins and BiP

**Figure 6:**
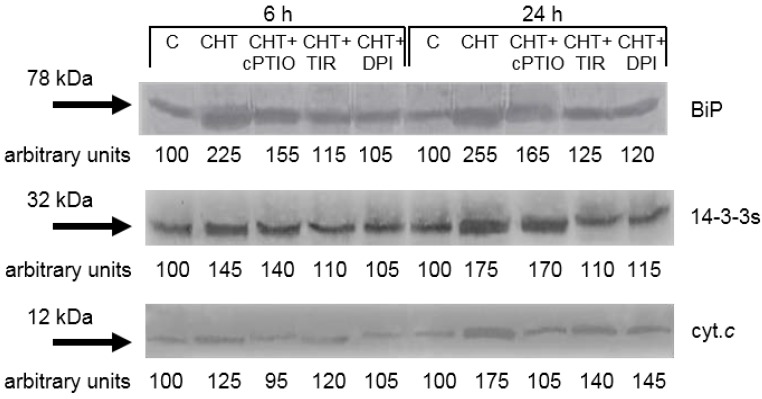
Effect of cPTIO, Tiron and DPI on the CHT-induced release of cytochrome *c* (cyc. *c*) from mitochondria and increase in the levels of cytosolic 14-3-3 proteins and BiP. C: Control cells; CHT: Cells treated with 0.005% CHT; CHT + Cptio: Cells treated with 50 μM cPTIO + 0.005% CHT; CHT + TIR: Cells treated with 2 μM Tiron + 0.005% CHT; CHT + DPI: Cells treated with 10 μM DPI + 0.005% CHT. The results of a typical experiment (*n* = 3) run in duplicate are shown. 50 μg of proteins were run in each lane. At each experimental time an arbitrary value of 100 was assigned to the amount of immunodecorated protein in the control cells.

We previously reported that CHT induces release of cytochrome *c* from the mitochondrion as well as increase in the levels of cytosolic 14-3-3 proteins and BiP [16]. The release of cytochrome *c* from the mitochondrion to the cytosol is one of the typical markers of cell death with apoptotic features in both animals and plants [21], The 14-3-3 proteins are a class of regulatory proteins playing a role in many processes of plant cells, including cytochrome *c* release and cell death [24], and BiP is a widely distributed and highly conserved HSP70 ER-resident molecular chaperone, which accumulates during different stresses [25]. The results presented in Figure 6 reconfirm the stimulatory effects of CHT on cytochrome *c* release and 14-3-3 proteins and BiP accumulation and show that: (i) The CHT-induced release of cytochrome *c* is totally prevented by the NO scavenger cPTIO, while Tiron and DPI are less effective; (ii) the CHT-induced accumulation of 14-3-3 proteins is totally prevented by Tiron and DPI, while cPTIO is ineffective; and (iii) the CHT-induced accumulation of BiP is reduced by cPTIO and almost completely prevented by Tiron and DPI.

### 2.6. Discussion

In a previous work we showed that in sycamore cells CHT induces, in addition to several defense/stress responses, the production of RNS and ROS [16]. It is known that these reactive species have a dual role in plants: they may act as toxic molecules leading to cell death from oxidative processes such as membrane lipid peroxidation, protein oxidation, enzyme inhibition and DNA and RNA damage, or may be involved as important signals in the regulation of many biological processes, PCD included [20,26]. In order to investigate whether the observed CHT-induced NO and ROS (namely O_2_•^−^ and H_2_O_2_) productions could be regarded as a mere symptom of cellular stress or whether they may have a signaling role, in this work we tested the effects of cPTIO, a specific NO scavenger, Tiron, a specific scavenger of superoxide anion (O_2_•^−^), and DPI a specific inhibitor of the NADPH oxidase (O_2_•^−^ synthase) of the plasma membrane on some defense/stress responses induced by CHT in sycamore cells: production of NO, O_2_•^−^, and H_2_O_2_; accumulation of dead cells and of cells with fragmented DNA; increase in the activity of caspase-3-like proteases; increase in lipid peroxidation; release of cytochrome *c* from the mitochondrion; accumulation of regulative 14-3-3 proteins in the cytosol and of HSP70 molecular chaperone BiP in the ER.

The NO scavenger cPTIO prevents the accumulation of dead cells and of cells with fragmented DNA, the increase in caspase-3-like activity and the release of cytochrome *c* from the mitochondrion induced by CHT. Otherwise, cPTIO is less effective in preventing the increase in lipid peroxidation and the accumulation of BiP while it is practically ineffective in preventing the accumulation of 14-3-3 proteins induced by CHT (Figure 3, Figure 4, Figure 5 and Figure 6). Although in plants a rigorous definition of cell death processes is still missing [27], in a previous work conducted with the same experimental material used in the present study we proposed that CHT activates different cell death programs. In particular, we proposed that low concentrations of CHT induce a cell death that could be regarded as an apoptotic-like process, while higher concentrations of CHT also induce a cell death that does not show the typical hallmarks of apoptosis [16]. This view is in agreement with studies performed using CHT in soybean and tobacco cultured cells [5,28]. Anyway, it should be considered that CHT, depending both on its concentration and its physiochemical properties (deacetylation degree, molecular weight and viscosity), can activate PCD or another form of cell death. In other words, there exists a threshold concentration for each CHT type able to switch controlled responses into cytotoxicity, that should be evaluated for each considered plant model [10]. The inhibitory effect of cPTIO on accumulation of TUNEL-positive cells, increase of caspase-3-like proteases and release of cytochrome *c* from the mitochondrion observed in this work led us to hypothesize the involvement of NO in a form ofCHT-induced cell death that presents apoptotic features. In the recent past, various studies on plant-microbe interaction have implicated NO as a signal molecule involved in several defense mechanisms of plants, particularly for biotic or abiotic stress management using an inducer/elicitor [20]. Involvement of NO in plant defense responses was substantiated through a pharmacological and genetical approach by altering NO levels [29] and NO is also known to activate the transcriptional factors of pathogenesis-related protein during the induction of resistance, which plays an important role in avoiding the pathogen advancement [30]. The role of NO in regulating hypersensitive reaction in plants associated with pathogen interaction is very well established [31]. Further, the direct control of NO over *Arabidopsis* metacaspase 9, a potential executioner of programmed cell death functionally analogous to typical caspases involved in apoptosis of animal cells, support a central role of NO in an apoptotic-like form of cell death of plants [32].

Tiron and DPI, inhibitors of ROS production, reduce the accumulation of dead cells, the increase in lipid peroxidation, the accumulation of 14-3-3 proteins and BiP induced by CHT. On the contrary, the two chemicals are not able to prevent the accumulation of cells with fragmented DNA, the increase in caspase-3-like activity, and the release of cytochrome *c* from the mitochondrion induced by CHT (Figure 3, Figure 4, Figure 5 and Figure 6).

Since the inherent toxic nature of ROS masked their underlying function in various signaling networks, the involvement of these molecules as a signal during different plant processes was, for a long time, rather hypothetical. But now it is accepted that plants can regulate the levels of antioxidant enzymes to purposefully use ROS as important signals in the induction and execution of several plant processes, cell death included [26,33]. ROS production has been observed in several plant systems challenged with CHT and their regulative role in different CHT-induced defense responses, cell death included, has been clearly elucidated [10]. In sycamore cells challenged with CHT, ROS seem involved in a form of cell death that does not show the typical apoptotic features but presents membrane lipid peroxidation. A possible alternative explanation of this ROS action might be a direct role of CHT on transcription when DNA is the primary target of CHT. This action on DNA could regulate the transcription of stress genes, primarily PR genes, which actually form the basis of disease resistance in plants [3]. In this case ROS would eventually act indirectly and this can also explain the lack of collateral damage by CHT until many hours after its application.

## 3. Experimental Section

### 3.1. Cell Culture Growth and Experimental Conditions

Sycamore* (Acer pseudoplatanus* L.) cultured cells (a kind gift of R. Bligny, Laboratoires de Physiologie Végétale de CNRS-INRA, Grenoble, France), a material suitable for biochemical and physiological studies because of the homogeneity and the relative small dimension of the cell clumps, were maintained in a White’s modified medium supplemented with 58.4 mM sucrose, 2.97 μM thiamine-HCl and 4 μM 2,4-dichlorophenoxyacetic acid as previously described [34]. Cells from cultures in the exponential phase of growth (7-day-old cultures) were utilized in the experiments. The cells were gathered by gentle centrifugation (2 min at 300× *g*) and resuspended in fresh culture medium at a final density of 10^6^ cells∙mL^−1^.

A stock solution of 0.5% chitosan (low molecular weight, batch MKBD3830 with deacetylation degree 92.2% and viscosity 42 cps; Sigma-Aldrich, cat. nr. 448869; St. Louis, MO, USA) in 0.5% aqueous acetic acid was freshly prepared, adjusting the pH to 5.6 with 0.1 N NaOH. CHT was added to the cell suspension at a final concentration of 0.005% while control samples were supplemented with 0.005% acetic acid adjusted to pH 5.6 with 0.1 N NaOH. These additions did not modify the pH of the culture medium. Cells challenged with culture medium containing this percentage of aqueous acetic acid did not show differences in the parameters considered in this work compared to cells without any addition [16] (data not shown).

cPTIO (50 μM final concentration) (Sigma-Aldrich, St. Louis, MO, USA), Tiron (2 μM final concentration), or DPI (10 μM final concentration), were added to the cell suspension 10 min before the start of experiments (addition of acetic acid or CHT). The addition of cPTIO, Tiron or DPI did not influence the parameters of the control cells considered in this work [35,36] (data not shown).

### 3.2. O_2_•^−^ and H_2_O_2_ Assays and NO Imaging

O_2_•^−^ anion generation by sycamore cells was measured as reduction of XTT (2,3-*bis*(2-methoxy-4-nitro-5-sulfophenyl)-5-[(phenylamino) carbonyl]-2*H*-tetrazolium hydroxide) to XTT formazan as described [37]. Briefly, 0.5 mM XTT was added to the cultures at 0 h and at the indicated times 1 mL aliquots of culture medium were collected and the absorbance value due to the formation of XTT formazan was determined at 470 nm by a Jasco V-530 spectrophotometer (Jasco Corporation, Tokyo, Japan). Immediately after the measure the culture medium was reintroduced in the culture flasks. The quantity of XTT formazan was determined using the molar extinction coefficient of 2.16 × 10^−4^ M^−1^∙cm^−1^[37].

H_2_O_2_ accumulation in the culture medium was measured by the xylenol orange colorimetric assay as previously described [38,39]. Briefly, at the indicated times 500 μL aliquots of culture medium were added to 500 μL of reaction mixture containing 500 μM ammonium ferrous sulphate, 50 mM H_2_SO_4_, 200 μM xylenol orange, 200 mM sorbitol. After 45 min at room temperature, the absorbance value was determined at 560 nm by a Jasco V-530 spectrophotometer. The amount of H_2_O_2_ was calculated from a standard curve obtained adding known amounts of H_2_O_2_ to 500 μL of the culture medium.

NO in the cells was visualized by the NO-reactive cell-permeant fluorescent probe 4-amino-5-methylamino-2',7'-difluorofluorescein (DAF-FM) diacetate (Alexis Biochemicals, Lausen, Switzerland), as previously described [39]. This cell-permeant probe, after deacetylation by cell esterases, reacts with NO to make a covalent triazole compound (DAF-FM triazole) which emits a bright green fluorescence [40]. The cells, collected by gentle centrifugation (1 min at 500× *g*), were resuspended in 10 μM DAF-FM diacetate. After 15 min of incubation at room temperature, the cells were observed with a Nikon Eclipse 90i fluorescence microscope equipped with fluorescein isothiocyanate (FITC) filter sets (Nikon, Tokyo, Japan).

### 3.3. Cell Viability Assay

Cell viability was determined by 10 min incubation of cell suspension (1 mL) with an equal volume of 150 μg∙mL^−1^ Evan’s Blue solution in distilled water. The excess of dye was removed from the suspension by repeated washing with deionized water, whereas the dye bound by dead cells was solubilized by boiling (5 min, 100 °C) the gathered (2 min at 300× *g*) cells in 50% aqueous methanol containing 2% SDS. The solubilized dye was separated from the cells by centrifugation (5 min at 1000× *g*) and quantified spectrophotometrically by measuring the absorbance at 600 nm with a Jasco V-530 spectrophotometer. Boiled cells (10 min, 100 °C) were used as control of 100% cell death [41].

### 3.4. TUNEL Procedure

To detect nuclear DNA fragmentation, the cells were treated with the terminal deoxynucleotidyl transferase-mediated dUTP nick end labelling procedure (TUNEL) with the fluorescein-dUTP-based cell death detection kit (Medical & Biological Laboratories, Nagoya, Japan) as previously described [34]. At the end of the TUNEL procedure, the cells were counterstained with Hoechst 3342 (5 μg∙mL^−1^) in phosphate-buffered saline, PBS (0.15 M NaCl, 2.7 mM KCl, 1.2 mM KH_2_PO_4_, 6.5 mM Na_2_HPO_4_, pH 7.2), and analyzed with a Nikon Eclipse 90i microscope under UV-visible light. The percentage of Hoechst 3342-labeled nuclei that were positive for the TUNEL reaction was calculated from the observation of at least 1000 cells.

### 3.5. Activity of Caspase-3-Like Proteases

The activity of caspase-3-like proteases was measured with the caspase-3 colorimetric activity assay kit according to the manufacturer’s instructions (BioVision Research Products, Mountain View, CA, USA). Briefly, at the indicated times sycamore cells were collected from 1 mL of cell suspension by centrifugation (500*× g*, 1 min), resuspended in 500 μL of lysis buffer and homogenized on ice for 2 min at the maximal speed with an Ultra-Turrax T25 device (International PBI, Milan, Italy). After 10 min of incubation on ice, the homogenates were centrifuged (10,000*× g*, 5 min) and the supernatants were immediately used. The assay mixture was composed of 70 μL of supernatant, 20 μL of reaction buffer and 10 μL of substrate (Ac-DEVD-ρNA). After incubation at 37 °C for 2 h in the presence or absence of the specific inhibitor of caspase-3 proteases Ac-DEVD-CHO the absorbance of hydrolyzed ρNA was quantified at 405 nm using a Jasco V-530 spectrophotometer.

### 3.6. Level of Lipid Peroxidation

The level of lipid peroxidation was estimated by measuring the content of malondialdehyde (MDA), a secondary end product of the oxidation of polyunsaturated fatty acids as described [42]. The assay is based on the reaction of MDA with two molecules of thiobarbituric acid (TBA) via an acid-catalyzed nucleophilic-addition reaction and the spectrophotometric detection of the derived molecules. Frozen cells (0.2 g) were homogenized on ice for 5 min at maximum speed with an Ultra-Turrax T25 device, adding 0.8 mL of 100 mM Na-phosphate buffer (pH 8) containing 5 mM EDTA and 1 mL of 25% (*w*/*v*) TCA. The homogenates were centrifuged at 10,000× *g* for 15 min at 4 °C. The supernatants were immediately used for the assay of MDA. To quantify MDA, 250 µL of the supernatant was added to 1 mL of 0.5% (*w*/*v*) TBA in 20% (*w*/*v*) TCA. The mixture was heated at 95 °C for 30 min and then quickly cooled on ice for 5 min. After centrifugation at 10,000× *g* for 10 min, the absorbance of the supernatant was measured at 532 nm by a Jasco V-530 spectrophotometer. The value for the non-specific absorbance at 600 nm was subtracted from the absorbance at 532 nm and the concentration of MDA was calculated using the extinction coefficient of 155 mM^−1^∙cm^–1^[43].

### 3.7. Cell Fraction Preparation

Cells were collected by gentle centrifugation, frozen in liquid nitrogen and homogenized for 5 min at maximum speed with a Ultra-Turrax T25 device at a density of 1 g of fresh weight per 2 mL of homogenizing buffer (25 mM 2-(*N*-morpholino)ethanesulfonic acid-2-(*bis*(2-hydroxymethyl)amino)-2-(hydroxymethyl)-1,3-propanediol, pH 7.8, 250 mM sucrose, 5 mM EDTA, 0.2% bovine serum albumin, and 0.2% casein) freshly supplied with 2 mM dithiothreitol and 10 μL of plant protease inhibitor cocktail (Sigma-Aldrich cat. nr. P9599) per mL of cell homogenate. The homogenate was centrifuged at 1000× *g* for 10 min and the supernatant was centrifuged again at 10,000× *g* for 15 min. The supernatant was centrifuged at 48,000× *g* for 60 min and the resultant pellet, representing the microsomal fraction, was resuspended in 10 mM Tris-HCl, pH 6.5, 1 mM EDTA, 1 mM dithiothreitol and 20% (*v*/*v*) glycerol supplemented with the protease inhibitor cocktail, and stored at −80 °C until use. The supernatant was centrifuged at 200,000× *g* for 3 h and the resultant supernatant, representing the cytosolic (soluble) fraction, was stored at −80 °C until use. All the above reported procedures were performed at 4 °C.

### 3.8. SDS-PAGE and Protein Gel Blot Analysis

Equal amounts of proteins, determined with the Bio-Rad microassay (Hercules, CA, USA) using bovine serum albumin as standard, were separated by discontinuous SDS-PAGE (4% stacking, 10% resolving gel), performed as described previously [44] in a Mini Protean II apparatus (Bio-Rad). The proteins were electrotransferred onto polyvinylidene difluoride membranes (Immobilon-P, Millipore Corporation, Billerica, MA, USA) using a Bio-Rad Mini Gel Trans Blot cell and immunodecorated. Immunodecoration of cytochrome *c* was performed on the cytosolic (soluble) fraction with a polyclonal antibody against full-length cytochrome *c* from horse heart (Santa Cruz Biotechnology, Santa Cruz, CA, USA). Immunodecoration of 14-3-3 proteins was performed on the cytosolic (soluble) fraction with an antibody against the brain modulosignalin homologue 1 (BMH1) isoform of yeast, a generous gift from Paul van Heusden, Leiden University, the Netherlands. Immunodecoration of BiP was performed on the microsomal fraction with an antibody against tobacco BiP, a generous gift from A. Vitale, Istituto di Biologia e Biotecnologia Agraria, CNR, Milano, Italy. The relative abundance of immunodecorated proteins was quantified using ImageJ software (version 1.32J; National Institutes of Health, Bethesda, MD, USA).

### 3.9. Statistical Analyses

Statistical analyses were performed using GraphPad Prism 4 program from GraphPad Software, Inc., San Diego, CA, USA.

## 4. Conclusions

Summarizing the results of this study show that both reactive nitrogen and oxygen species are not only a mere symptom of stress conditions but are involved in the responses induced by CHT in sycamore cells. In particular, the inhibiting effect of cPTIO on the CHT-induced accumulation of cells with fragmented DNA, increase in caspase-3-like activity and release of cytochrome *c* from the mitochondrion suggests the involvement of NO in a cell death form induced by CHT that shows these apoptotic features. On the contrary, the inhibiting effect of Tiron and DPI on the CHT-induced cell death together with the lack of effect on the CHT-induced accumulation of cells with fragmented DNA, increase in caspase-3-like activity and release of cytochrome *c* from the mitochondrion suggests the involvement of ROS in a cell death form induced by CHT that does not show these apoptotic features but presents increase in lipid peroxidation. Both types of reactive species seem to be involved in accumulation of BiP while only ROS seem to be involved in the accumulation of 14-3-3 proteins. The relative small effect of cPTIO in preventing BiP accumulation may be regarded as a symptom of an involvement in this response of molecules like peroxynitrite, a reactive nitrogen species formed when NO reacts with O_2_•^−^. In fact, even though peroxynitrite is not involved in NO-mediated cell death, it is emerging as a potential signaling molecule during the induction of defense responses against pathogens by the selective nitration of tyrosine residues in a small number of proteins [45].

## Abbreviation

BiP: Binding Protein
CHT: Chitosan
DAF-FM: 4-Amino-5-methylamino-2',7'-difluorofluorescein
DiOC_6_: 3,3'-Dihexyloxacarbocyanine iodide
ER: Endoplasmic reticulum
NO: Nitric oxide
PCD: Programmed cell death
ROS: Reactive oxygen species
RNS: Reactive nitrogen species
TUNEL: Terminal deoxynucleotidyl transferase-mediated dUTP nick end labeling
XTT: 2,3-*Bis*-(2-Methoxy-4-nitro-5-sulfophenyl)-2*H*-tetrazolium-5-carboxanilide
Tiron: 4,5-Dihydroxy-1,3-benzene disulfonic acid
DPI: Diphenylene iodonium
cPTIO: 2-(4-Carboxyphenyl)-4,4,5,5-tetramethylimidazoline-1-oxyl-3-oxide
